# Stories for a sustainable healthcare future: the perspectives of healthcare IoT technologies in surgical oncology

**DOI:** 10.25122/jml-2023-0198

**Published:** 2023-05

**Authors:** Ciprian Ianovici, Victor Lorin Purcărea, Iuliana-Raluca Gheorghe, Alexandru Blidaru

**Affiliations:** 1. Department of Health Care Marketing and Medical Technology, Carol Davila University of Medicine and Pharmacy, Bucharest, Romania; 2. Department of Surgical Oncology, Carol Davila University of Medicine and Pharmacy, Bucharest, Romania

**Keywords:** IoT technologies, surgical oncology, sustainable healthcare

## Abstract

The rapid spread and continuous development of Internet of Things (IoT) technologies have profoundly impacted various aspects of daily life, including healthcare. The aim of this review was to highlight the necessity of adopting healthcare IoT technologies in surgical oncology clinical practice, remote home monitoring of cancer patients, and context awareness facilitation. In addition, we identified different categories of healthcare IoT technologies and the best practice examples of IoT technologies for breast cancer patients. Moreover, discussions were supported by the future opportunities of integrating IoT technologies in surgical oncology.

## CURRENT APPLICATION OF HEALTHCARE IoT TECHNOLOGIES

In recent years, there have been a growing acceptance and utilization of Internet of Things (IoT) technologies in various domains, such as manufacturing, transportation, and agriculture [1], but also in healthcare. The necessity of finding solutions to ease the strain of healthcare systems while maintaining the provision of health quality care, with the help of the Internet of Things (IoT) technologies, is already acknowledged [2-4]. Although the use of IoT technologies in healthcare is still in its early stages, their potential application in several medical scenarios has become increasingly relevant [5]. For instance, even before the pandemic, healthcare systems worldwide struggled to provide quality healthcare services to patients, and healthcare professionals were looking for solutions that would potentially lower the expenses associated with the constantly aging population and the growth associated with chronic illnesses [5].

Today, there exists a convergence of Information Communication Technology (ICT) infrastructure, hardware, software, and networking technologies in healthcare, aiming to connect different stakeholders such as patients, physicians, healthcare providers, health data repositories, medical centers, and hospitals in a shared information environment [6]. The need for this connected environment stems from the continuous rehabilitation and monitoring of the patients [7-9]. The advantages of highly connected stakeholders include improved clinical and patient safety, reduced spread of contagious diseases, and increased clinical productivity by providing accurate and timely information [10]. However, in practice, stakeholders are not well-connected, which leads to a serious number of flaws that are spread across the healthcare system [11].

At an individual level, IoT technology has a significant impact on raising people’s health awareness and improving and monitoring their health statuses [12]. As such, IoT technology is changing the healthcare industry by completely reinventing the devices, applications, and people involved in healthcare solutions [13], and the industry is continually producing new tools to guarantee better patient care [14] by offering remote home monitoring. Despite not being widely used in all aspects of the healthcare industry, the adoption is exponential, even in the clinical setting, and is already being integrated into cardiology, nutrition, and neurology [15]. The use of IoT technology in healthcare aims to streamline daily procedures for healthcare providers and assess the quality of patient care and satisfaction. Similarly, IoT technology can track patients, manage oncology medical inventories, and facilitate mental treatments [16-18]. Therefore, IoT technologies have the potential to be utilized in clinical care, remote monitoring, and situational awareness [15].

The automatic medical data gathering method lowers the chance of human error data collection, increasing the accuracy of the diagnosis and lowering the possibility of human mistakes [19]. IoT technologies in clinical care focus on enabling constant observation and close monitoring of patients to respond to potential crises and save lives. These monitoring systems use sensors to gather physiological data, which is processed, stored, and delivered online to caregivers. By collaborating and analyzing the data collected by sensors, multiple healthcare professionals can collectively provide treatment services based on their respective areas of expertise.

Remote monitoring emphasizes real-time data collection, even for children, older individuals, or patients with chronic illnesses [20]. The remote access of sensors enables the early detection and interventions of caregivers, but there is also the possibility for persons with various cognitive and physical impairments to live more independently and comfortably [21]. As such, the sensor can be used to diagnose heart conditions and the impact of medications on their health and actions. For instance, patients with chronic conditions like cardiopulmonary disease, asthma, and heart failure may use wireless monitoring systems based on sensors to collect information and determine real-time processing of the sensor data. Consequently, the Wireless Local Area Network (WLAN) system transmits the sensor’s body signals to the appropriate medical facility.

Context awareness, or situational awareness, highlights the importance of understanding a patient's health status based on various factors such as their activity patterns (e.g., walking, running, or sleeping) or the environment they reside in (e.g., wet, cold, hot). With the help of IoT technologies, physicians can determine the likelihood of a patient developing certain diseases based on the collected information over time. This information enables physicians to identify when a patient requires urgent assistance and, importantly, what immediate actions need to be taken.

## EXAMPLES OF HEALTHCARE IoT TECHNOLOGY PROJECTS

Although IoT technology systems were created for various healthcare objectives, they are all closely related because they use the same enabling technologies [5]. An example of IoT applicability was the tracking of Parkinson’s disease [18]. According to the researchers, wearable sensors may detect tremors and patterns in patients with Parkinson’s disease, which can provide valuable insights into the disease progression. The IoT wearable device may be accompanied by vision-based technologies, such as cameras around the patient’s home.

Another example relates to a heart attack tracking device with a readily available and unique antenna [22]. The heart activity of the user is measured by an electrocardiograph sensor and processed by a microprocessor. The ECG data is analyzed and displayed on the user’s mobile phone via Bluetooth connectivity. The heart attack prediction may also be assessed by measuring the respiratory rate.

Another helpful example of IoT technology in healthcare relates to patients discharged from the hospital [23]. This IoT system supports physicians in identifying potential complications and readmissions among these patients. The researchers involved in this IoT project emphasize the feasibility of safely extracting information from sensors and transferring it to a cloud-based platform. By doing so, timely suggestions can be made to mitigate the risk of patient readmission, as the first seven days following discharge are particularly critical. Cloud-based availability ensures data accuracy and safety.

SPHERE is one of the most advanced healthcare IoT technologies, which is still being developed [24]. SPHERE uses a vision-based approach with cameras, wearable devices, and environmental sensors to monitor general activity and the patient's health. The project’s goal is to monitor the health of older and chronically ill people, ensuring comfort in their homes. This IoT project also allows caregivers and medical professionals to intervene in real time. According to the researchers, the project is based on machine learning, which would help understand diseases and make judgments regarding the patients’ healthcare.

## TYPES OF HEALTHCARE IoT TECHNOLOGIES

The scientific literature identifies two major categories of healthcare IoT technologies that have the potential to address various challenges in clinical practice, remote patient monitoring and context awareness. These categories are healthcare IoT services and healthcare IoT applications [19]. The healthcare IoT services consist of protocols that may assist patients and physicians in detecting several medical conditions, in contrast with the healthcare IoT applications installed on different gadgets and devices, such as mobile phones, tablets, laptops, and wearable items. The distinction between these categories lies in their orientation, with healthcare IoT services being developer-centered and healthcare IoT applications being user-centered. Within these categories, several subcategories of healthcare IoT technologies are identified [19]:

a. Healthcare IoT services
  - Ambient Assisted Living (AAL) services focus on providing older and disabled individuals with an independent lifestyle while ensuring their safety.
  - Internet of e-Health Things, or mobile IoT, involves using mobile computers and sensor technology for context awareness, often sharing data with 4G networks.
  - Adverse Drug Reaction is a side effect overdosing identifier, which is based on a patient’s electronic health record, and can check the medication by scanning the barcode of a medicine.
  - Community health services focus on the medical status of a community, such as a neighborhood, school, hospital, etc. These services are provided through cooperative networks with proper authorization mechanisms.
  - Children's health information services aim to educate, empower, and assist in pediatric care by monitoring the behavioral, emotional, and mental health development of children.
  - Indirect emergency healthcare services focus on providing solutions in emergency circumstances such as weather calamities, transportation accidents, and fires.
b. Healthcare IoT applications
  - Glucose level sensing devices provide real-time monitoring and sensing for individuals with diabetes or older individuals, enabling accurate glucose level measurements and organization of activities and medication schedules.
  - IoT-based electrocardiogram (ECG) monitoring devices track the cardiovascular system by measuring heart rate, identifying rhythm irregularities, and detecting myocardial and ischemic heart diseases. These devices incorporate real-time monitoring and search automation for normal and pathological cardiac activities.
  - The blood pressure monitoring device aims to keep track of blood pressure levels and consists of a blood pressure kit and a mobile phone that serves as an IoT-based blood pressure system.
  - The body temperature monitoring device includes a body sensor monitoring system that provides precise, effective, and successful temperature assessment based on infrared detection.
  - The oxygen saturation monitoring device is similar to the pulse oximeter device, but, being a wearable device, it can continuously monitor blood oxygen saturation. The sensor’s information is collected with an IoT-optimized low-power and low-cost pulse and is transmitted via Bluetooth to a smartphone.
  - Rehabilitation systems for older and physically impaired patients focus on efficiently implementing remote consultations and real-time information collection.
  - The IoT-based medication management consists of intelligent packaging of pharmaceutical boxes for patients based on prescriptions from physicians, and it connects electronic health records with AAL solutions.
  - IoT wheelchair management aims to provide full automation for disabled individuals, utilizing various sensors to collect data about the patients' environments for localized assessments.
  - Prompt medical solutions. Despite the availability of many portable medical devices, there is no concrete integration into IoT networks. Extensive research is required, but due to the increased demand in IoT based services, several medical gadgets and IoT applications are expected to be adopted in medical practice and even be recommended for patients after remote surgery, for cancer therapy, or for patients who suffer from eye disorders, abnormal cellular growth, peak expiratory flow, and skin infections. In these cases, prompt medical solutions and accessibility to medical consultation or information are paramount.

## THE CASE OF THE IoT TECHNOLOGY ADOPTION IN SURGICAL ONCOLOGY

Cancer is a global health concern and a leading cause of death worldwide [25]. Many cancer patients also experience other chronic diseases or related symptoms, such as dyslipidemia, diabetes, osteoporosis, depression, anxiety, weight loss, and anemia [26-29]. The treatment options can vary depending on the stage of the disease and may include surgery, immunotherapy, chemotherapy, radiotherapy, and hormone replacement [30]. Being such a complex disease, innovative IoT technology devices may be useful in supporting patients during and after the indicated treatment to minimize the impact on their quality of life as much as possible. IoT technology has shown promising applications in predicting readmission [31] and facilitating recovery [32] for cancer patients, especially after surgery.

IoT physical activity trackers have been used to monitor the physical activity of cancer patients discharged after surgery [33]. There are various examples of effective physical activity trackers, such as Activate [34], which use actigraphy technology to obtain behavioral feedback and coaching sessions for stage I-III breast cancer patients. Some researchers claim that the possible behavioral changes may be attributed to IoT technologies by providing more education to users on the matter or wearable devices [35]. In a prospective study involving 65 breast cancer patients, it was noted that 16.9% of them never used a wearable IoT device, suggesting that an easy-to-use IoT is required for some persons [36].

Other IoT devices for cancer patients should monitor the quality-of-life parameters, also known as biometric parameters [37]. Several IoT application devices for monitoring the heart rate, blood pressure, weight, and specific symptoms should be included, but there are wearable devices that measure the depression levels of cancer patients [38].

Sensor networks connected to machine learning algorithms have been developed to track the risk of different cancers. For instance, the iTBra IoT project for breast cancer allows for the monitoring of specific symptoms associated with the development of breast cancer, aiding in early detection [39].

While the relevance of IoT technology in clinical practice is still being validated, existing literature on wearable devices and IoT technologies by cancer patients has shown positive feedback, particularly in the case of breast cancer survivors [40]. For example, Nguyen et al. [41] showed that breast cancer patients accepted wearing physical activity trackers, thus having efficient outcomes in preventing sedentary behaviors.

Despite the positive IoT technology acceptance by patients, the information collected by wearable devices and tracking sensors has not yet been integrated into a system, and some of the IoT technologies still need clinical validation [6].

## FUTURE HEALTHCARE OPPORTUNITIES FOR IoT TECHNOLOGY IN SURGICAL ONCOLOGY

Nowadays, IoT technology gadgets and wearable devices cannot be integrated into a network to which physicians and patients have access and are usually limited to a single physician-patient health monitoring. However, there are several future opportunities for implementing healthcare IoT technologies, particularly in clinical practice [6]. In the clinical setting, IoT support for healthcare can involve the implementation of architecture and platforms that enable clinicians and health professionals to respond quickly and effectively to patients' medical needs, whether remotely or in person. This can reduce the number of appointments required and support prioritizing cases that require urgent assistance. In addition, by using remote surgery, it is possible to provide high-quality healthcare to underdeveloped regions that do not have easy access to a healthcare system. As such, IoT remote surgery is made possible by several technologies, including a wireless and satellite communication channel, machine learning, imaging technology, artificial intelligence, and tele-surgical robots. Such IoT-based solutions can be used in a healthcare system, either independently or in conjunction with professionals.

## CONCLUSION

The integration of IoT technologies in healthcare systems represents a significant milestone in the future of the medical field as it offers the potential to create more connected and data-driven healthcare services. The advantages of implementing IoT technologies in surgical oncology relate to keeping cancer patients better informed, involved, and empowered with information, which enables them to actively participate in maintaining a sustainable healthcare system. For other healthcare stakeholders, IoT technologies may provide accurate and ready-to-use information, as well as facilitate their interaction and exchange of expertise with other stakeholders. However, the full potential of IoT technologies can be realized if patients are encouraged to play a participatory role in the learning process and are actively involved in all stages of IoT system development.
